# Di-μ-azido-κ^4^ *N* ^1^:*N* ^1′^-bis­({1-[(*E*)-phen­yl(pyridin-2-yl-κ*N*)methyl­idene]thio­semi­carbazidato-κ^2^ *N* ^1^,*S*}copper(II))

**DOI:** 10.1107/S1600536812035751

**Published:** 2012-08-23

**Authors:** Roji J. Kunnath, M. R. Prathapachandra Kurup, Seik Weng Ng

**Affiliations:** a Department of Applied Chemistry, Cochin University of Science and Technology, Kochi 682 022, India; b Department of Chemistry, University of Malaya, 50603 Kuala Lumpur, Malaysia; c Chemistry Department, King Abdulaziz University, PO Box 80203 Jeddah, Saudi Arabia

## Abstract

In the title compound, [Cu_2_(C_13_H_11_N_4_S)_2_(N_3_)_2_], the Cu^II^ cation is *N*,*N*′,*S*-chelated by the deprotonated Schiff base ligand and is coordinated by the azide anion, while an N atom from an adjacent azide anion bridges the Cu^II^ cation at the apical position with a longer Cu—N distance of 2.533 (3) Å, completing the distorted N_4_S square-pyramidal coordination geometry. A pair of azide anions bridge the two Cu^II^ cations, forming a centrosymmetric binuclear mol­ecule. In the crystal, the binuclear mol­ecules are linked by an N—H⋯N hydrogen bond into a ribbon running along the *a* axis.

## Related literature

For the structure of the parent Schiff base, see: Casas *et al.* (2003 ▶).
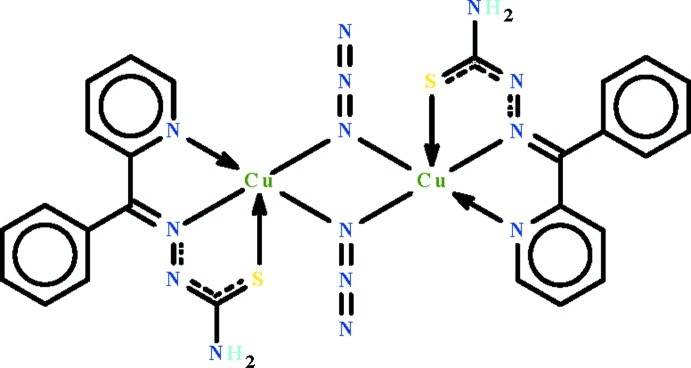


## Experimental

### 

#### Crystal data

[Cu_2_(C_13_H_11_N_4_S)_2_(N_3_)_2_]
*M* *_r_* = 721.78
Monoclinic, 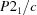

*a* = 11.2462 (12) Å
*b* = 7.2344 (10) Å
*c* = 18.519 (2) Å
β = 96.653 (5)°
*V* = 1496.5 (3) Å^3^
*Z* = 2
Mo *K*α radiation
μ = 1.61 mm^−1^
*T* = 295 K
0.35 × 0.30 × 0.25 mm

#### Data collection

Bruker Kappa APEXII diffractometer
Absorption correction: multi-scan (*SADABS*; Sheldrick, 1996 ▶) *T*_min_ = 0.604, *T*_max_ = 0.690
13614 measured reflections
3747 independent reflections
2973 reflections with *I* > 2σ(*I*)
*R*_int_ = 0.075

#### Refinement

*R*[*F* ^2^ > 2σ(*F* ^2^)] = 0.045
*wR*(*F* ^2^) = 0.134
*S* = 1.04
3747 reflections
205 parameters
2 restraints
H atoms treated by a mixture of independent and constrained refinement
Δρ_max_ = 0.54 e Å^−3^
Δρ_min_ = −0.66 e Å^−3^

### 

Data collection: *APEX2* (Bruker, 2010 ▶); cell refinement: *SAINT* (Bruker, 2010 ▶); data reduction: *SAINT*; program(s) used to solve structure: *SHELXS97* (Sheldrick, 2008 ▶); program(s) used to refine structure: *SHELXL97* (Sheldrick, 2008 ▶); molecular graphics: *X-SEED* (Barbour, 2001 ▶); software used to prepare material for publication: *publCIF* (Westrip, 2010 ▶).

## Supplementary Material

Crystal structure: contains datablock(s) global, I. DOI: 10.1107/S1600536812035751/xu5611sup1.cif

Structure factors: contains datablock(s) I. DOI: 10.1107/S1600536812035751/xu5611Isup2.hkl

Additional supplementary materials:  crystallographic information; 3D view; checkCIF report

## Figures and Tables

**Table 1:** Hydrogen-bond geometry (Å, °)

*D*—H⋯*A*	*D*—H	H⋯*A*	*D*⋯*A*	*D*—H⋯*A*
N3—H1⋯N2^i^	0.87 (1)	2.22 (1)	3.075 (3)	168 (4)

Symmetry code: (i) 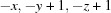.

**Fig. 1.**
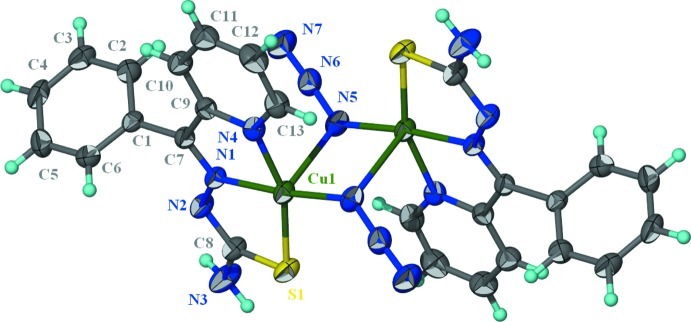
Thermal ellipsoid plot (Barbour, 2001) of [Cu(N3)(C13H11N4S)]2 at the 570% probability level; hydrogen atoms are drawn as spheres of arbitrary radius.

## supplementary crystallographic information

### Comment

2-Benzoylpyridine thiosemicarbazone (Casas *et al.*, 2003) is a Schiff base that is capable of *N*,*N*',*S*-chelation to metal ions. The Cu^II^ atom in [Cu(N_3_)(C_13_H_11_N_4_S)]_2_ (Scheme I) is *N*,*N*',*S*-chelated by the deprotonated Schiff base, and it exists in a square pyramidal environment (Fig. 1). Two molecules are disposed about a center-of-inversion and the distance between the copper atom and their apical nitrogen atom of the other azide is 2.533 (3) Å. Adjacent inversion-related pairs of molecules are linked by an N–H···N hydrogen bond to form a ribbon running along the *a*-axis (Table 1).

### Experimental

The Schiff base ligand by heating 2-benzoylpyridine (0.183 g,1 mmol) and thiosemicarbazide (0.091 g,1 mmol) for 3 h. Copper acetate hydrate (0.199 g,1 mmol) and sodium azide (0.065 g,1 mmol) was added and the solution heated for another 2 h. Dark green colored crystals were obtained from the cool solution.

### Refinement

Carbon-bound H-atoms were placed in calculated positions (C–H 0.93 Å) and were included in the refinement in the riding model approximation, with *U*(H) set to 1.2*U*(C).

The amino H-atoms were located in a difference Fouier and were refined with a distance restraint of N–H 0.88±0.01 Å; their temperature factors tied by a factor of 1.2 times.

Omitted owing interference from the beam stop was (1 0 0).

### Crystal data

**Table:**

[Cu_2_(C_13_H_11_N_4_S)_2_(N_3_)_2_]	*F*(000) = 732
*M**_r_* = 721.78	*D*_x_ = 1.602 Mg m^−^^3^
Monoclinic, *P*2_1_/*c*	Mo *K*α radiation, λ = 0.71073 Å
Hall symbol: -P 2ybc	Cell parameters from 4663 reflections
*a* = 11.2462 (12) Å	θ = 3.0–28.3°
*b* = 7.2344 (10) Å	µ = 1.61 mm^−^^1^
*c* = 18.519 (2) Å	*T* = 295 K
β = 96.653 (5)°	Prism, dark green
*V* = 1496.5 (3) Å^3^	0.35 × 0.30 × 0.25 mm
*Z* = 2	

### Data collection

**Table:**

Bruker Kappa APEXII diffractometer	3747 independent reflections
Radiation source: fine-focus sealed tube	2973 reflections with *I* > 2σ(*I*)
Graphite monochromator	*R*_int_ = 0.075
ω scans	θ_max_ = 28.4°, θ_min_ = 2.2°
Absorption correction: multi-scan (*SADABS*; Sheldrick, 1996)	*h* = −14→15
*T*_min_ = 0.604, *T*_max_ = 0.690	*k* = −9→9
13614 measured reflections	*l* = −24→24

### Refinement

**Table:**

Refinement on *F*^2^	Primary atom site location: structure-invariant direct methods
Least-squares matrix: full	Secondary atom site location: difference Fourier map
*R*[*F*^2^ > 2σ(*F*^2^)] = 0.045	Hydrogen site location: inferred from neighbouring sites
*wR*(*F*^2^) = 0.134	H atoms treated by a mixture of independent and constrained refinement
*S* = 1.04	*w* = 1/[σ^2^(*F*_o_^2^) + (0.0694*P*)^2^ + 0.1161*P*] where *P* = (*F*_o_^2^ + 2*F*_c_^2^)/3
3747 reflections	(Δ/σ)_max_ = 0.001
205 parameters	Δρ_max_ = 0.54 e Å^−^^3^
2 restraints	Δρ_min_ = −0.66 e Å^−^^3^

### Fractional atomic coordinates and isotropic or equivalent isotropic displacement parameters (Å^2^)

**Table:**

	*x*	*y*	*z*	*U*_iso_*/*U*_eq_	
Cu1	0.40816 (2)	0.66511 (5)	0.521018 (15)	0.03737 (14)	
S1	0.31362 (6)	0.55753 (11)	0.61376 (3)	0.0469 (2)	
N1	0.24572 (17)	0.7151 (3)	0.47503 (10)	0.0345 (4)	
N2	0.14750 (19)	0.6402 (3)	0.50005 (12)	0.0408 (5)	
N3	0.0841 (2)	0.4802 (5)	0.59265 (14)	0.0617 (8)	
H1	0.023 (2)	0.456 (6)	0.5610 (15)	0.074*	
H2	0.101 (3)	0.422 (5)	0.6343 (12)	0.074*	
N4	0.44641 (18)	0.8236 (3)	0.43679 (11)	0.0377 (5)	
N5	0.5749 (2)	0.6199 (4)	0.55657 (12)	0.0468 (6)	
N6	0.6113 (2)	0.5803 (4)	0.61823 (13)	0.0487 (6)	
N7	0.6497 (3)	0.5408 (5)	0.67545 (14)	0.0721 (9)	
C1	0.1174 (2)	0.8573 (3)	0.37564 (12)	0.0340 (5)	
C2	0.0861 (3)	0.7823 (4)	0.30754 (14)	0.0454 (6)	
H2A	0.1412	0.7109	0.2861	0.054*	
C3	−0.0264 (3)	0.8127 (5)	0.27110 (15)	0.0506 (7)	
H3	−0.0470	0.7627	0.2251	0.061*	
C4	−0.1078 (2)	0.9171 (5)	0.30306 (16)	0.0506 (7)	
H4	−0.1848	0.9339	0.2796	0.061*	
C5	−0.0752 (3)	0.9963 (5)	0.36967 (17)	0.0556 (8)	
H5	−0.1294	1.0707	0.3906	0.067*	
C6	0.0364 (2)	0.9669 (4)	0.40563 (14)	0.0456 (6)	
H6	0.0576	1.0216	0.4507	0.055*	
C7	0.2345 (2)	0.8130 (3)	0.41657 (12)	0.0335 (5)	
C8	0.1738 (2)	0.5609 (4)	0.56389 (13)	0.0414 (6)	
C9	0.3482 (2)	0.8757 (4)	0.39278 (13)	0.0349 (5)	
C10	0.3555 (2)	0.9761 (4)	0.33033 (14)	0.0438 (6)	
H10	0.2866	1.0115	0.3010	0.053*	
C11	0.4675 (3)	1.0232 (5)	0.31207 (16)	0.0510 (7)	
H11	0.4750	1.0910	0.2702	0.061*	
C12	0.5668 (3)	0.9688 (5)	0.35637 (17)	0.0546 (7)	
H12	0.6428	0.9978	0.3446	0.065*	
C13	0.5534 (2)	0.8708 (4)	0.41863 (16)	0.0460 (6)	
H13	0.6214	0.8364	0.4490	0.055*	

### Atomic displacement parameters (Å^2^)

**Table:**

	*U*^11^	*U*^22^	*U*^33^	*U*^12^	*U*^13^	*U*^23^
Cu1	0.03127 (19)	0.0431 (2)	0.0358 (2)	0.00031 (11)	−0.00433 (13)	0.00303 (12)
S1	0.0438 (4)	0.0589 (5)	0.0356 (3)	−0.0033 (3)	−0.0049 (3)	0.0100 (3)
N1	0.0312 (9)	0.0386 (12)	0.0328 (9)	−0.0013 (8)	−0.0002 (7)	0.0049 (8)
N2	0.0332 (10)	0.0478 (14)	0.0404 (11)	−0.0046 (9)	−0.0003 (8)	0.0132 (9)
N3	0.0541 (15)	0.079 (2)	0.0504 (14)	−0.0192 (14)	−0.0032 (11)	0.0287 (14)
N4	0.0325 (10)	0.0390 (13)	0.0410 (11)	−0.0018 (8)	0.0018 (8)	−0.0002 (8)
N5	0.0366 (11)	0.0595 (16)	0.0412 (12)	0.0036 (11)	−0.0092 (9)	0.0006 (11)
N6	0.0408 (11)	0.0518 (16)	0.0499 (13)	0.0046 (10)	−0.0099 (10)	−0.0094 (11)
N7	0.081 (2)	0.083 (2)	0.0462 (14)	0.0153 (16)	−0.0196 (13)	−0.0044 (14)
C1	0.0341 (11)	0.0368 (14)	0.0308 (11)	−0.0008 (9)	0.0026 (9)	0.0077 (9)
C2	0.0456 (14)	0.0489 (17)	0.0402 (13)	0.0035 (12)	−0.0013 (10)	−0.0058 (12)
C3	0.0508 (16)	0.061 (2)	0.0370 (13)	−0.0032 (13)	−0.0064 (11)	0.0039 (12)
C4	0.0367 (13)	0.060 (2)	0.0527 (15)	0.0040 (12)	−0.0052 (11)	0.0219 (14)
C5	0.0421 (14)	0.065 (2)	0.0608 (17)	0.0200 (14)	0.0120 (12)	0.0086 (15)
C6	0.0455 (14)	0.0547 (18)	0.0368 (12)	0.0050 (12)	0.0057 (10)	0.0012 (11)
C7	0.0319 (11)	0.0343 (14)	0.0337 (11)	0.0022 (9)	0.0012 (9)	0.0002 (9)
C8	0.0409 (13)	0.0449 (17)	0.0370 (12)	−0.0025 (11)	−0.0006 (10)	0.0075 (10)
C9	0.0348 (11)	0.0332 (13)	0.0369 (11)	−0.0001 (10)	0.0050 (9)	−0.0004 (9)
C10	0.0446 (14)	0.0453 (17)	0.0418 (13)	−0.0020 (11)	0.0062 (11)	0.0050 (11)
C11	0.0551 (17)	0.0497 (18)	0.0508 (15)	−0.0051 (13)	0.0171 (13)	0.0057 (13)
C12	0.0425 (14)	0.055 (2)	0.0695 (19)	−0.0090 (13)	0.0208 (13)	−0.0018 (15)
C13	0.0336 (12)	0.0482 (17)	0.0557 (16)	−0.0032 (11)	0.0034 (11)	−0.0029 (12)

### Geometric parameters (Å, º)

**Table:**

Cu1—N5	1.942 (2)	C2—C3	1.381 (4)
Cu1—N1	1.9578 (19)	C2—H2A	0.9300
Cu1—N4	2.022 (2)	C3—C4	1.373 (4)
Cu1—S1	2.2603 (8)	C3—H3	0.9300
Cu1—N5^i^	2.533 (3)	C4—C5	1.371 (5)
S1—C8	1.729 (3)	C4—H4	0.9300
N1—C7	1.287 (3)	C5—C6	1.368 (4)
N1—N2	1.359 (3)	C5—H5	0.9300
N2—C8	1.316 (3)	C6—H6	0.9300
N3—C8	1.329 (4)	C7—C9	1.472 (3)
N3—H1	0.872 (10)	C9—C10	1.376 (3)
N3—H2	0.879 (10)	C10—C11	1.384 (4)
N4—C13	1.331 (3)	C10—H10	0.9300
N4—C9	1.348 (3)	C11—C12	1.364 (4)
N5—N6	1.202 (3)	C11—H11	0.9300
N6—N7	1.134 (3)	C12—C13	1.377 (4)
C1—C6	1.372 (4)	C12—H12	0.9300
C1—C2	1.380 (4)	C13—H13	0.9300
C1—C7	1.477 (3)		
			
N5—Cu1—N1	173.91 (9)	C2—C3—H3	120.1
N5—Cu1—N4	94.22 (9)	C5—C4—C3	119.7 (2)
N1—Cu1—N4	80.26 (8)	C5—C4—H4	120.1
N5—Cu1—S1	101.84 (7)	C3—C4—H4	120.1
N1—Cu1—S1	84.08 (6)	C6—C5—C4	120.5 (3)
N4—Cu1—S1	160.44 (7)	C6—C5—H5	119.7
N5—Cu1—N5^i^	85.54 (9)	C4—C5—H5	119.7
N1—Cu1—N5^i^	91.79 (8)	C5—C6—C1	120.5 (3)
N4—Cu1—N5^i^	89.28 (8)	C5—C6—H6	119.8
S1—Cu1—N5^i^	102.92 (6)	C1—C6—H6	119.8
C8—S1—Cu1	93.90 (9)	N1—C7—C9	114.7 (2)
C7—N1—N2	120.1 (2)	N1—C7—C1	123.1 (2)
C7—N1—Cu1	117.58 (17)	C9—C7—C1	122.3 (2)
N2—N1—Cu1	122.15 (15)	N2—C8—N3	116.7 (2)
C8—N2—N1	111.9 (2)	N2—C8—S1	125.6 (2)
C8—N3—H1	114 (2)	N3—C8—S1	117.71 (19)
C8—N3—H2	118 (3)	N4—C9—C10	122.1 (2)
H1—N3—H2	124 (4)	N4—C9—C7	114.3 (2)
C13—N4—C9	118.5 (2)	C10—C9—C7	123.6 (2)
C13—N4—Cu1	128.30 (19)	C9—C10—C11	118.7 (3)
C9—N4—Cu1	113.11 (16)	C9—C10—H10	120.7
N6—N5—Cu1	124.8 (2)	C11—C10—H10	120.7
N7—N6—N5	177.3 (3)	C12—C11—C10	119.1 (3)
C6—C1—C2	119.1 (2)	C12—C11—H11	120.4
C6—C1—C7	120.8 (2)	C10—C11—H11	120.4
C2—C1—C7	120.1 (2)	C11—C12—C13	119.4 (3)
C1—C2—C3	120.4 (3)	C11—C12—H12	120.3
C1—C2—H2A	119.8	C13—C12—H12	120.3
C3—C2—H2A	119.8	N4—C13—C12	122.2 (3)
C4—C3—C2	119.8 (3)	N4—C13—H13	118.9
C4—C3—H3	120.1	C12—C13—H13	118.9
			
N5—Cu1—S1—C8	−167.21 (13)	C2—C1—C6—C5	2.2 (4)
N1—Cu1—S1—C8	11.35 (12)	C7—C1—C6—C5	−175.2 (3)
N4—Cu1—S1—C8	48.2 (2)	N2—N1—C7—C9	−176.1 (2)
N5^i^—Cu1—S1—C8	−79.13 (11)	Cu1—N1—C7—C9	−0.5 (3)
N4—Cu1—N1—C7	1.37 (19)	N2—N1—C7—C1	3.0 (4)
S1—Cu1—N1—C7	169.6 (2)	Cu1—N1—C7—C1	178.59 (18)
N5^i^—Cu1—N1—C7	−87.6 (2)	C6—C1—C7—N1	65.7 (4)
N4—Cu1—N1—N2	176.9 (2)	C2—C1—C7—N1	−111.7 (3)
S1—Cu1—N1—N2	−14.88 (19)	C6—C1—C7—C9	−115.3 (3)
N5^i^—Cu1—N1—N2	87.9 (2)	C2—C1—C7—C9	67.3 (3)
C7—N1—N2—C8	−173.5 (2)	N1—N2—C8—N3	−178.8 (3)
Cu1—N1—N2—C8	11.1 (3)	N1—N2—C8—S1	2.6 (4)
N5—Cu1—N4—C13	−0.4 (2)	Cu1—S1—C8—N2	−11.5 (3)
N1—Cu1—N4—C13	−177.8 (3)	Cu1—S1—C8—N3	170.0 (3)
S1—Cu1—N4—C13	144.9 (2)	C13—N4—C9—C10	−0.3 (4)
N5^i^—Cu1—N4—C13	−85.9 (2)	Cu1—N4—C9—C10	−176.6 (2)
N5—Cu1—N4—C9	175.44 (18)	C13—N4—C9—C7	178.6 (2)
N1—Cu1—N4—C9	−1.97 (17)	Cu1—N4—C9—C7	2.2 (3)
S1—Cu1—N4—C9	−39.2 (3)	N1—C7—C9—N4	−1.2 (3)
N5^i^—Cu1—N4—C9	89.97 (18)	C1—C7—C9—N4	179.7 (2)
N4—Cu1—N5—N6	155.8 (3)	N1—C7—C9—C10	177.6 (3)
S1—Cu1—N5—N6	−13.0 (3)	C1—C7—C9—C10	−1.5 (4)
N5^i^—Cu1—N5—N6	−115.3 (3)	N4—C9—C10—C11	0.6 (4)
C6—C1—C2—C3	−2.0 (4)	C7—C9—C10—C11	−178.1 (3)
C7—C1—C2—C3	175.5 (3)	C9—C10—C11—C12	0.0 (5)
C1—C2—C3—C4	−0.5 (5)	C10—C11—C12—C13	−0.9 (5)
C2—C3—C4—C5	2.6 (5)	C9—N4—C13—C12	−0.7 (4)
C3—C4—C5—C6	−2.3 (5)	Cu1—N4—C13—C12	175.0 (2)
C4—C5—C6—C1	−0.1 (5)	C11—C12—C13—N4	1.3 (5)

Symmetry code: (i) −*x*+1, −*y*+1, −*z*+1.

### Hydrogen-bond geometry (Å, º)

**Table:**

*D*—H···*A*	*D*—H	H···*A*	*D*···*A*	*D*—H···*A*
N3—H1···N2^ii^	0.87 (1)	2.22 (1)	3.075 (3)	168 (4)

Symmetry code: (ii) −*x*, −*y*+1, −*z*+1.
